# Metabolism of AGEs – Bacterial AGEs Are Degraded by Metallo-Proteases

**DOI:** 10.1371/journal.pone.0074970

**Published:** 2013-10-09

**Authors:** Ifat Cohen-Or, Chen Katz, Eliora Z. Ron

**Affiliations:** 1 Department of Molecular Microbiology and Biotechnology, Life Sciences, Tel Aviv University, Tel Aviv, Israel; 2 MIGAL – Galilee Research Center, Kiriat Shmone, Israel; Oak Ridge National Laboratory, United States of America

## Abstract

Advanced Glycation End Products (AGEs) are the final products of non-enzymatic protein glycation that results in loss of protein structure and function. We have previously shown that in *E. coli* AGEs are continually formed as high-molecular weight protein complexes. Moreover, we showed that AGEs are removed from the cells by an active, ATP-dependent secretion and that these secreted molecules have low molecular weight. Taken together, these results indicate that *E. coli* contains a fraction of low molecular weight AGEs, in addition to the high-molecular weight AGEs. Here we show that the low-molecular weight AGEs originate from high-molecular weight AGEs by proteolytic degradation. Results of *in-vitro* and *in vivo* experiments indicated that this degradation is carried out not by the major ATP-dependent proteases that are responsible for the main part of bacterial protein quality control but by an alternative metal-dependent proteolysis. This proteolytic reaction is essential for the further secretion of AGEs from the cells. As the biochemical reactions involving AGEs are not yet understood, the implication of a metalloprotease in breakdown of high molecular weight AGEs and their secretion constitutes an important step in the understanding of AGEs metabolism.

## Introduction

Advanced Glycation End-products (AGEs) are the final products of non-enzymatic glycation formed by the reaction of reactive carbonyls (e.g.- reducing sugars) with primary amine-containing amino acids of proteins. One of the first steps in this glycation process is the formation of Amadori-modified proteins (AMPs) which are reversible intermediates. These AMPs can further developed, in an oxidation-dependent manner, to form advanced protein complexes, that contain irreversible, highly stable high molecular weight AGEs [1–4] . Although AGEs can be formed by a direct interaction of sugar metabolites and free amino acids, in the cells, where the concentration of free amino acids is fairly low, the major fraction of AGEs is probably formed as a subsequent metabolic step from glycation-modified proteins [5]. 

 In mammals, AGEs were shown to accumulate both intracellularly and extracellularly with age and to participate in the pathophysiology of several age-related diseases such as cardiovascular disease, Alzheimer’s disease and complications of diabetes mellitus [6–10]. They accumulate in many sites, including the kidney, retina, and atherosclerotic plaques [11] and their toxic effects in mammalian models were extensively studied [12–14].

Cells maintain the quality and functionality of proteins by degradation and replacement of damaged proteins. Although glycation is one of the most common types of physiological protein damages, very little is known about the protein quality control mechanisms that participate in their metabolism. In humans, AGEs were found to be released into blood plasma and excreted in urine, with the kidney as the major site of AGE clearance. Studying the physiological effect of inducible glycation stress has shown that treatment of cells with the glycating agent - glyoxal - resulted in cessation of proteasome activity both *in-vivo* and *in-vitro* but did not affect degradation of AGEs, suggesting that AGEs are not degraded by the cellular proteasome [15,16]. It was also shown that the extracellular AGEs are more resistant to enzymatic degradation probably due to their tendency to aggregate and it is likely that this property promotes local accumulation of AGEs in several tissues [11,15,17,18].

 Ineffective clearance of AGEs leads to their accumulation and consequent damage [11,17,19,20]. Therefore, understanding the metabolism of AGEs and pathways involving their secretion is essential. The secreted AGEs have lower molecular weight than the AGEs in the tissues. Clearly, then, there must be a degradative step that leads to the formation of the smaller molecules. However, to the best of our knowledge, intracellular proteolysis of endogenous glycated proteins has never been demonstrated, and a specific mechanism of AGEs proteolysis has not been identified so far [17,19]. 

Thus, it appears that while the effect of AGEs on mammalian physiology has been extensively studied, very little is known about their metabolism. We have recently proposed the use of bacteria as a novel tool for the study of AGEs metabolism. We provided evidences that glycated proteins are metabolized in bacteria and that low-molecular weight AGEs are actively secreted by bacteria into the growth media [21] [22]. In bacteria, formation of AGEs is restricted to the high molecular protein fraction [21]. However, we demonstrated that AGEs are also found as low-molecular-weight molecules and it is in this form that they are secreted from the cells. 

In this work, we investigated the proteolytic stage that precede AGEs secretion and show, both *in-vitro* and *in-vivo*, that metallo-proteases are involved in AGEs degradation. We also show that AGEs degradation is a required step for AGEs secretion, as inhibition the degradation results in reduced secretion and accumulation of AGEs inside the bacterial cell. These findings further our understanding of AGEs metabolism and can shed a light on the general mechanisms responsible for AGEs degradation in bacteria and possibly other organisms

## Results

### Bacterial AGEs are degraded by a metallo protease

**Figure 1:**
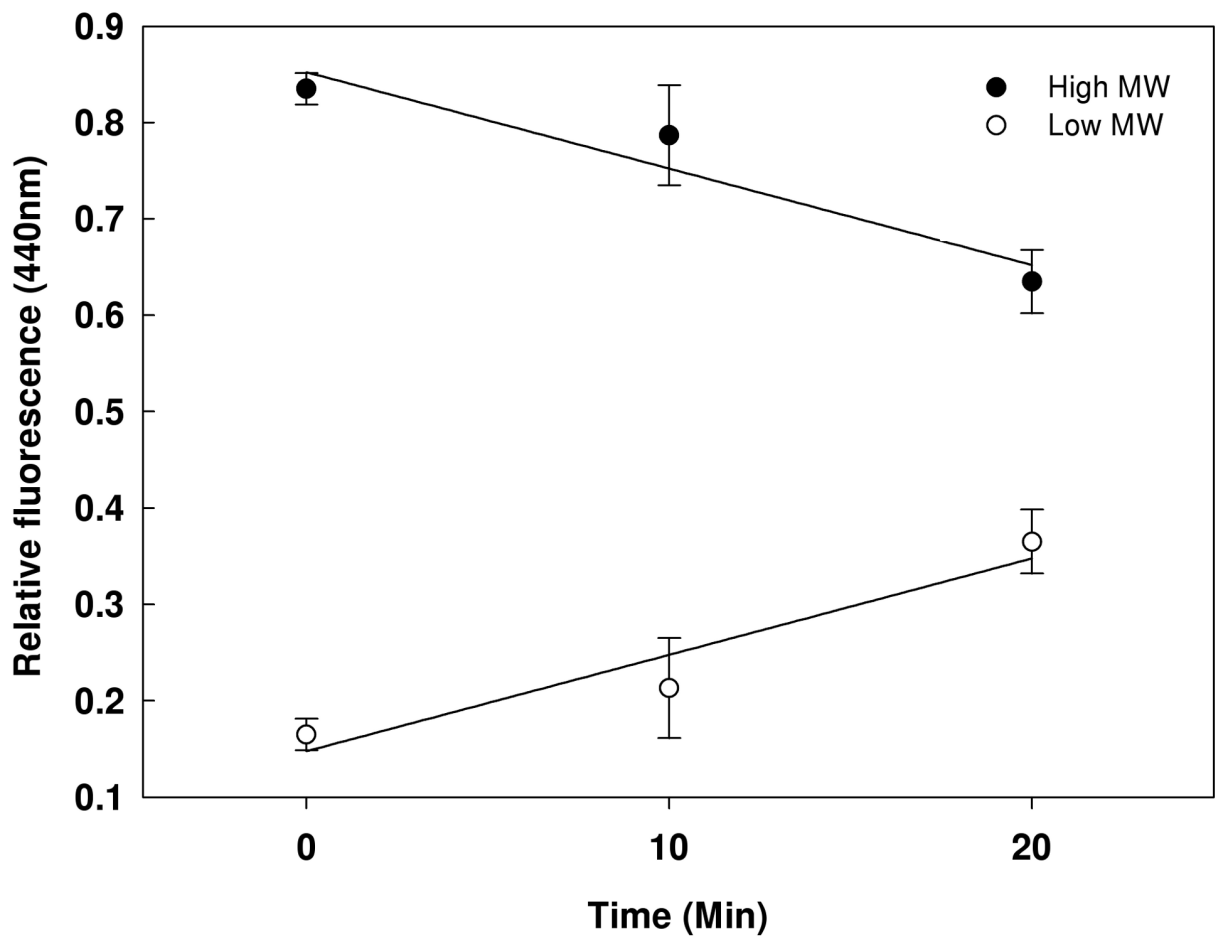
Effect of arsenate and chloramphenicol on AGEs size profile. Lysates were extracted from exponentially growing cultures at time intervals after addition of arsenate and chloramphenicol. Samples were separated into proteins and low molecular weight compounds fractions, as described in Materials and Methods. AGEs-specific fluorescence was monitored and normalized to cell density. AGEs level in the high molecular weight proteins fraction (full circles) and low-molecular weight fractions (empty circles).

The finding that AGEs – formed as high molecular weight compounds - are also found in low-molecular weight fractions [21] leads to the hypothesis that AGEs are proteolytically degraded. To study AGEs degradation *in-vivo* we arrested protein translation and AGEs secretion, using chloramphenicol and arsenate [22], and measured the concentration of intracellular AGEs specific fluorescence both in the high and in the low-molecular-weight fractions. In the beginning of the experiment (representing the steady state of AGEs in the cells) less than 20% of AGEs were found as low-molecular-weight compounds. However, following the arrest in protein synthesis and AGEs secretion there was a significant increase of small AGEs that reached about 40% of the total AGEs after 20 minutes (Figure 1). 

**Figure 2:**
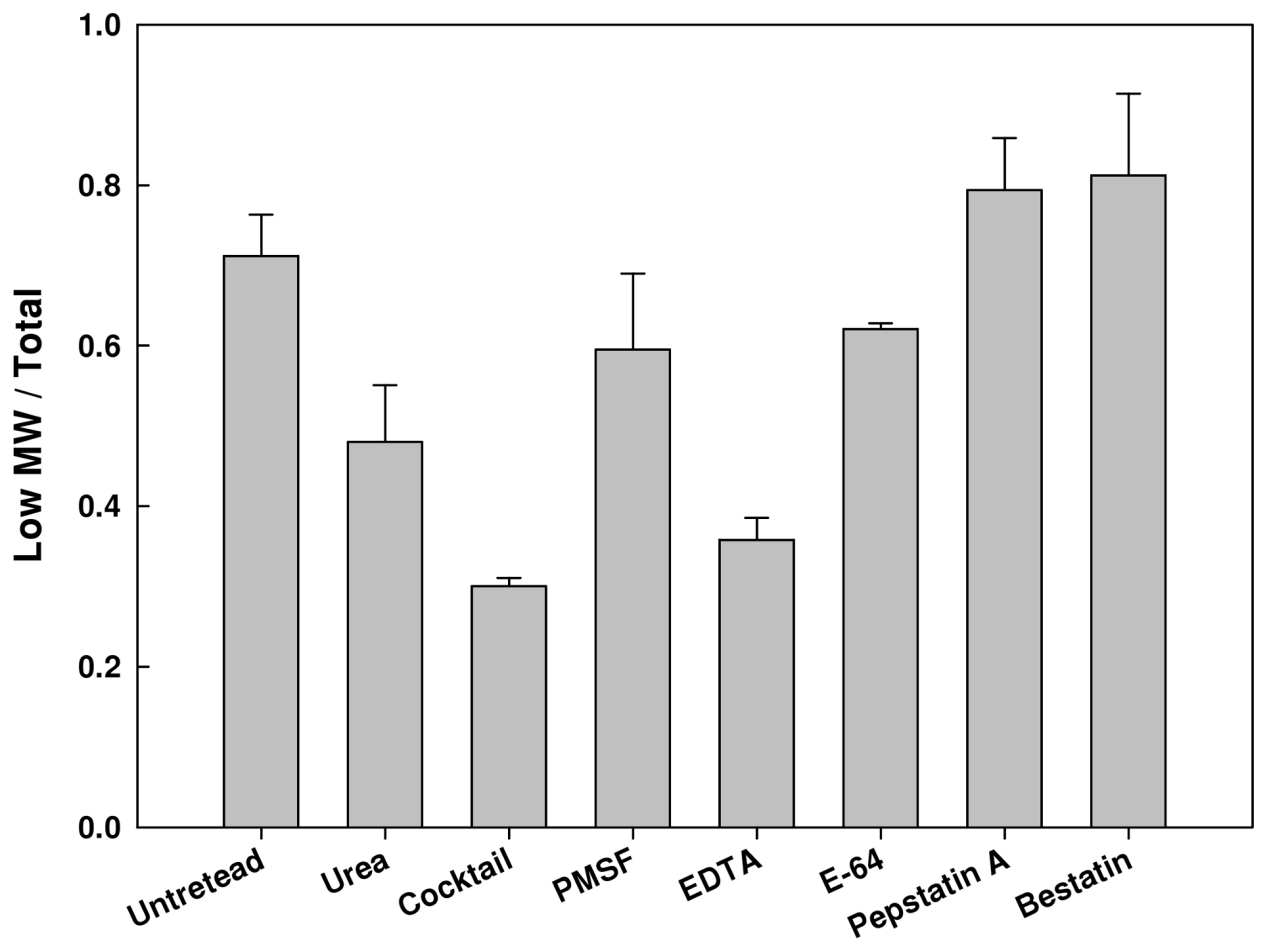
AGEs degradation *in-vitro*. Lysates were extracted from exponentially growing cultures and separated into high and low-molecular-mass fractions, as described in Materials and Methods. The high molecular weight fraction was incubated at 37°C for 3 hours with various protease inhibitors and then separated again into high and low-molecular-mass fractions. AGE-specific fluorescence was determined. The graph represents the percentage of low-molecular-mass fraction from the total AGEs in an untreated sample. The treatments were with 3M urea, protease inhibitor cocktail (Sigma), 25 mM MPSF (serine-protease inhibitor), 10 mM EDTA (metallo-protease inhibitor), 0.3 mM E-64 (cystein-protease inhibitor), 0.3 mM Pepstatin A (aspartate -protease inhibitor) and 2 mM Bestatin (amino-protease inhibitor).

In order to study the mechanism of AGEs degradation we developed an *in-vitro* assay in which we determined the kinetics of AGEs degradation under several experimental conditions. High-molecular-weight protein fractions were incubated at 37°C, fractionated, and the levels of high and low molecular-weight AGEs were measured. After 3 hours of incubation the majority (~70%) of AGEs were proteolytically cleaved to low molecular-weight compounds (Figure 2). However, upon the addition of 3M urea degradation level was significantly reduced, indicating that this degradation requires protein activity. Similar results were obtained following the addition of a protease inhibitor cocktail, further supporting the assumption that proteolytic activity is responsible for the degradation of AGEs.

Next we examined the possible involvement of several families of proteases by selective inhibition of their activity. We added inhibitors of serine-proteases, metallo-proteases, cystein-proteases, aspartate-proteases and amino-proteases. The results, displayed in Figure 2, pointed out that the metallo-protease inhibitor had the most significant effect on AGEs cleavage, indicating that metallo-proteases are responsible for the majority of AGEs degradation.

**Figure 3:**
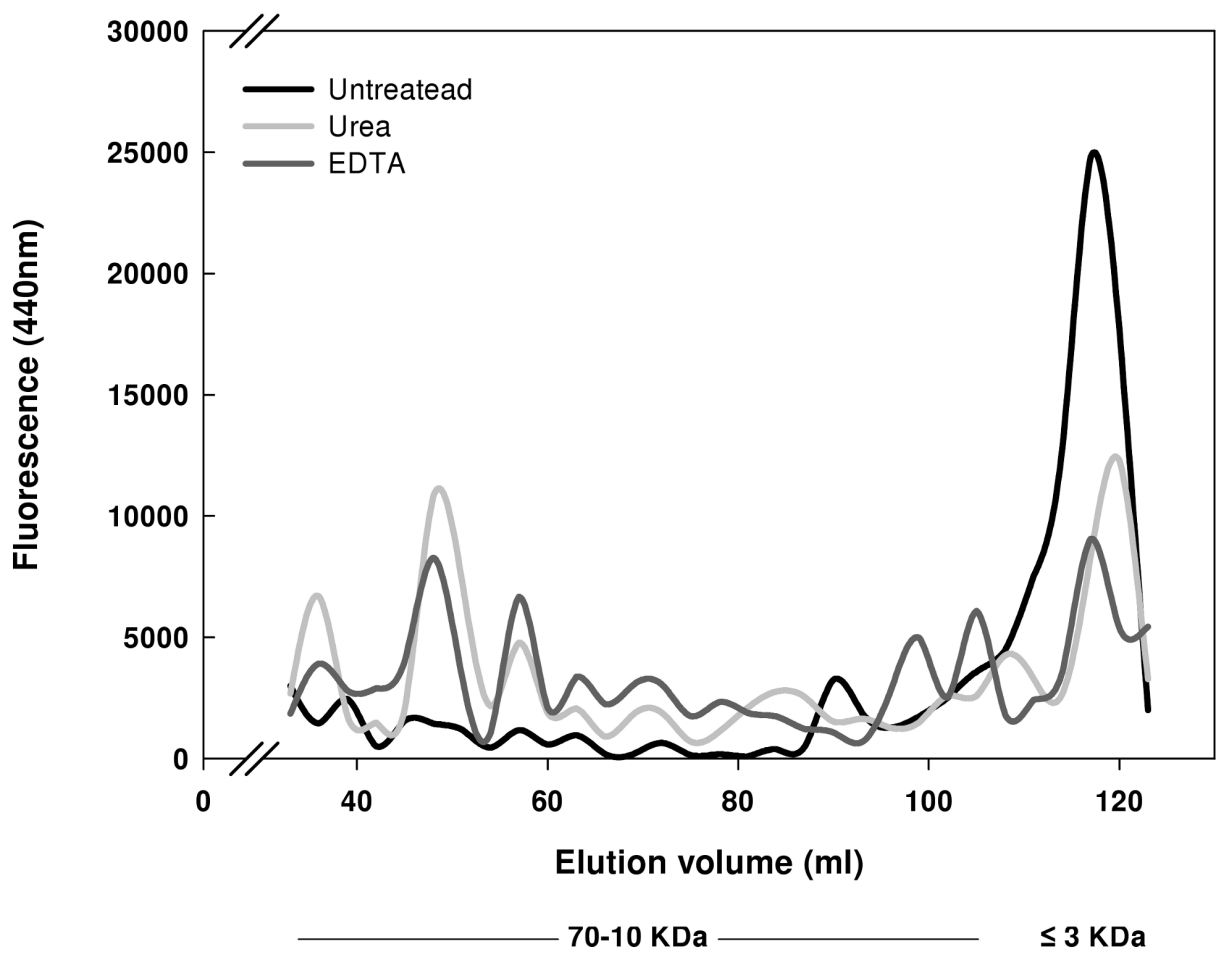
AGEs size exultation filtration *in-vitro*. Lysate was extracted from exponentially growing cultures and further separated into high and low-molecular-mass fractions, as described in Materials and Methods. The high molecular weight fraction was treated and incubated at 37°C for 3 hours and then separated by size using HiLoad 16/600 Superdex 75 pg 120 ml filtration column. After 30 ml of elution, 4 ml fractions were collected and AGEs specific fluorescence was determined. The graph represents the size distribution of AGEs in an untreated sample, lystae treated with 3M urea and lystae treated with protease inhibitor cocktail (Sigma).

In the experiments described in Figures 1 and 2 we analyzed AGEs in two fractions - low molecular weight and high molecular weight – corresponding to <5KD and >5KD respectively. However, in cells AGEs are found in a range of molecular weights and moreover the size of degradation intermediates can vary. Therefore, for a more accurate study it was important to examine the whole range of sizes. For this purpose we analyzed AGEs size with high resolution gel-filtration column. High-molecular weight fractions were obtained, using GE Healthcare HiTrap desalting columns, and incubated as before. After the incubation, samples were separated using high resolution gel filtration chromatography and fractions were collected and analyzed for AGEs specific fluorescence. In agreement with the pervious results, both the use of 3M urea and metallo-protease inhibitor exhibited a different AGEs size profile compared to an untreated sample (Figure 3). In both cases the relative amounts of high-molecular-weight AGEs were higher and the levels of low MW AGEs were reduced. These results strengthen the previous results and demonstrate that metallo-proteases have a significant involvement in the proteolytic degradation of AGEs. 

**Figure 4:**
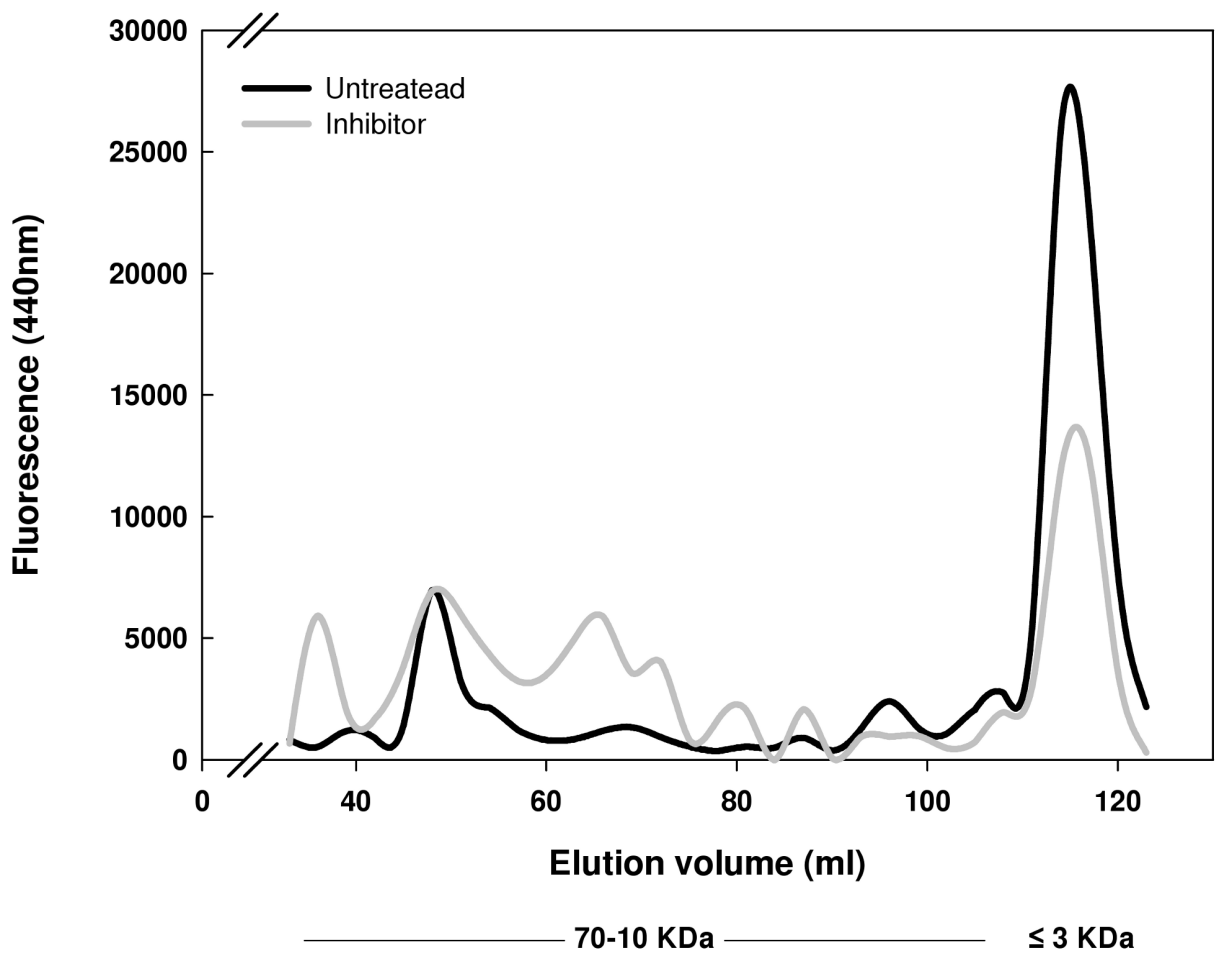
Size profile of AGEs following metallo-protease inhibition *in-vivo*. Effect of 100 µM 1,10 phenantholine (Sigma) on AGEs degradation. Bacteria were grown as described in materials and methods. Lysate was extracted from treated and untreated cells and further separated by size using HiLoad 16/600 Superdex 75 pg 120 ml filtration column. After 30 ml of elution, 4 ml fractions were collected and AGEs specific fluorescence was determined.

Next we examined the involvement of metallo-proteases *in-vivo*. Bacterial cultures were treated with the membrane permeable metallo-protease inhibitor -1,10 phenantholine [23–25]. After 10 minutes, samples were taken from both treated and untreated cultures, lysed and separated by size exclusion chromatography for determination of AGEs-specific fluorescence. The results presented in Figure 4 indicate that the treated cultures contain elevated amount of high-molecular-weight AGEs, as compared with the untreated samples. These results indicate that metallo-proteases affect AGEs cleavage also *in-vivo*, and their inhibition leads to accumulation of high-molecular-weight AGEs.

### AGEs degradation is required for secretion

**Figure 5:**
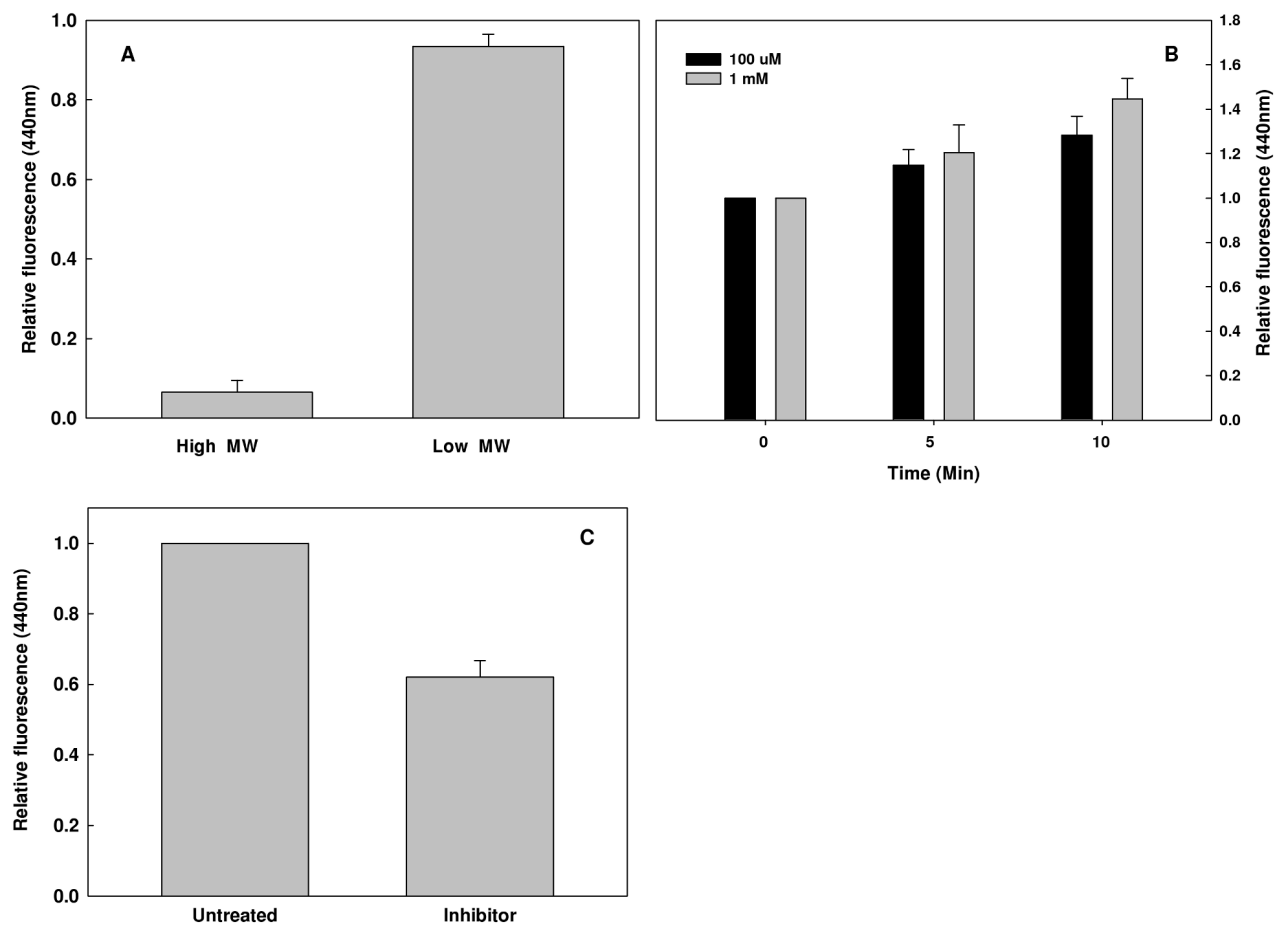
Effect of metallo-protease inhibition on AGEs accumulation and secretion. Bacterial growth and sample collection were described in materials and methods. AGEs-specific fluorescence was determined and normalized to cells density. A) Extracellular fraction was further separated into proteins and low molecular weight compounds fractions. B) Effect of 100 µM and 1 mM of 1,10 phenantholine (Sigma) on intracellular AGEs accumulation. C) Effect of 100 µM of 1,10 phenantholine (Sigma) on AGEs secretion after 2 hours in a non-growing cultures.

 Previously we showed that AGEs are secreted by bacteria [22]. In order to determine the size distribution of the secreted AGEs, extra-cellular fractions were collected and separated to low and high molecular-weight compounds. AGEs specific fluorescence signals were determined in each fraction. The results presented in Figure 5A demonstrate that at least 90% of the secreted AGEs molecules are of low molecular-weight. These results lead to the hypothesis that proteolytic degradation of AGEs is an early step that precedes AGEs secretion. In order to study the relationships between degradation and secretion of AGEs, we treated bacterial culture with 1,10 phenantholine – a metallo-protease inhibitor and examined its effect on the kinetics of AGEs secretion. Inhibition of AGEs degradation resulted in reduced secretion of AGEs (Figure 5B) and their accumulation inside the bacterial cell (Figure 5C). 

## Discussion

Advanced Glycation End-products (AGEs) are irreversible toxic molecules that accumulate in cells with time. Given their strong correlation to various diseases, the physiological effects of AGEs were extensively studied in human and conditions that affect the kinetics of their formation are well documented. However, pathways that participate in AGEs metabolism are poorly characterized and mechanisms that reduce their toxicity have not been extensively studied.

In mammals, it was found that AGEs are secreted from the body by the urinary system and that ineffective clearance of AGEs leads to their accumulation [11,22]. While the metabolism of extracellular AGEs (glycation of extracellular proteins) was fairly well studied, little is known about degradation of intracellular AGEs. Moreover, it is not yet clear whether the AGEs that are found in the blood stream originate from the metabolism of intracellular glycation products followed by their secretion, or restricted to extracellular glycation events. Additionally, although it appears that AGEs are degraded to lower molecular weight compounds, so far no specific proteolytic pathway has been identified [17,19].

Non-enzymatic glycation is a ubiquitous process, and therefore we have suggested that bacteria can constitute a powerful tool in the study of this complicated pathway. Here we showed that AGEs are proteolyticlly degraded inside *E. coli* cells and provided evidences that inside the bacterial cells high-molecular-weight AGEs are degraded to form low-molecular weight compounds. This degradation is attributed to the protein moiety of the AGEs as the total fluorescence signal of the AGEs remains the same while their size distribution changes with time. To our best knowledge, this is the first direct evidence for intracellular degradation of AGEs. 

*In-vitro* assays suggest that AGEs degradation is enzymatic, since denaturative agents such as urea and proteases inhibitor cocktail have reduced the rate of cleavage. Further examination on the various components of the proteases inhibitor cocktail revealed that metallo-proteases inhibitors are the most effective in prevention of AGEs degradation suggesting that metallo-proteases are the main players responsible for AGEs degradation. Similar observations were made *in-vivo*. 

These results indicate that the major ATP-dependent proteases, responsible for the main part of bacterial protein quality control, are not involved in the degradation of AGEs. Supporting this conclusion is the fact that Arsenate, a glycolysis uncoupler that severely reduces intracellular ATP concentration, did not affect the kinetics of AGEs degradation inside the bacterial cell. These results are compatible with the data obtained in eukaryotes suggesting that the proteasome, the eukaryotic equivalent to bacterial ATP-dependent proteases, is not responsible for proteolytic degradation of AGEs in higher organisms [15,16].

In *E. coli*, there are 30 metallo-proteases of which 5 are essential proteins. In an attempt to characterize the specific proteases that are involved in AGEs degradation we used mutants from the Keio knockout library collection [26], and analyzed the effect of each knock-out on the degradation kinetics. As inhibition of AGEs degradation results in accumulation of intracellular AGEs, we used this property to screen for relevant mutants. Unfortunately we could not find a single gene knockout that demonstrates a significant alternation of AGEs profile. The most significant change was observed in the *pqq*l deletion strain, lacking a putative zinc peptidase [27], which accumulates about 15% more AGEs as compared to the wild-type. However, this small effect, although reproducible, could not explain the significant impact of the metallo-proteases inhibitor. It is possible that there is a significant protease redundancy in AGEs degradation, so that a deletion in one protease is compensated by the presence of other proteases. Alternatively, it is possible to assume that a protease specific for AGEs degradation does exist, but has so far not been identified. 

We propose the following model; inside bacterial cells AGEs are mainly formed as high-molecular-weight modified proteins. Since AGEs are irreversible molecules they accumulate with time inside the cells. Clearance of these AGEs by secreting them is one strategy of the cells to cope with AGEs accumulation. However, as AGEs are not secreted as high molecular weight compounds, the protein moieties have to be degraded inside the cells prior to secretion. This degradation is probably performed by one or more metalloproteases. Unlike the major degradation pathways in *E. coli*, this degradation appears to be independent of energy, although the subsequent secretion of the low molecular weight AGEs is carried out by the energy-dependent efflux-pumps system [22]. At this point we are not able to support or to exclude the possibility that *in-vivo* the degradation and secretion are linked, but have shown that the degradation is required for the secretion. Thus, we propose that AGEs degradation is a preliminary step, necessary for the clearance of toxic AGEs.

Unlike other cellular damage, accumulation of AGEs presents an additional challenge to living organisms as AGEs can further react with other macromolecules such as lipids, proteins, and DNA leading to extensive damage potential [28]. On the other hand, aggregates of AGEs, once formed, are known to be highly resistant to enzymatic degradation [11,15,17,18]. These facts emphasize the importance of an efficient system for AGEs removal that will quickly degrade AGEs and prevent the formation of aggregates and secondary damages. The low molecular weight AGEs that are formed post degradation are highly soluble and can therefore be further removed from biological systems. We therefore hypothesize that this pathway of proteolytic degradation followed by the removal of low molecular weight AGEs may be conserved among organisms from all kingdoms.

## Materials and Methods

### Bacterial strains and growth conditions

The *E. coli* K12 wild type strain MG1655 was used throughout. Cultures were grown with aeration at 37°C in standard LB medium (Difco). When required cultures were treated with 100 µM / 1 mM 1,10 phenantholine (Sigma), 100 µg/ml chloramphenicol (Sigma) or 50 µg/ml arsenate (Sigma). 

The effect of 1,10 phenantholine on AGEs levels *in-vivo*, was measured shortly after the inhibitor was added, in order to minimize a bias caused by its effect on bacterial growth rate. The accumulation of AGEs shown in the first 10 minutes is followed by a reduction of total AGEs due to growth arrest

Determinations of the levels of secreted AGEs were performed in a non-growing culture using carbon-free media, as previously described [29].

### Sample preparation

Lysates were obtained using the Qproteome-bacterial Protein Prep kit (QIAGEN) according to the manufacturer's recommendation. The lysates were further separated into purified proteins and low molecular weight compounds using GE Healthcare HiTrap desalting columns, equilibrated with PBS, using Amersham AKTA prime plus FPLC system. These columns possess exclusion limit of 5000 g/mol of globular compound, thus compounds with lower molar mass are hereafter referred to as low molecular weight compounds. Nucleic acids were digested as part of Qproteome extraction procedure, and therefore were present mostly in the low molecular weight fraction. For AGEs size exclusion filtration measurements (Figure 3 and Figure 4) samples were separated using GE HiLoad 16/600 Superdex 75 pg 120ml filtration column, equilibrated with PBS. Separations were performed and evaluated using Amersham AKTA prime plus FPLC system. The first 30 ml was not collected; afterwards 4 ml fractions were collected.

### Determination of AGEs

AGEs were quantified using the natural AGE-specific fluorescence by scanning emission ranging from 400 nm to 500 nm upon excitation at 370 nm at 37°C, in a HORIBA scientific FluoroLog-3 Spectrofluorometer. Data represent the 440 nm emission peak (Ex. 370, Em. 440) 
